# Catechin gallate triggers metabolomic and lipidomic alteration in *Toxoplasma gondii*

**DOI:** 10.1186/s13071-025-06869-x

**Published:** 2025-07-31

**Authors:** Jazmyn R. Greggs, Homa Nath Sharma, Daniel A. Abugri

**Affiliations:** 1 Department of Biological Sciences, College of Science, Technology, Engineering and Mathematics, Alabama State University, Montgomery, AL 36104 USA; 2 Microbiology PhD Program, Department of Biological Sciences, College of Science, Technology, Engineering, and Mathematics, Alabama State University, Montgomery, AL 36104 USA; 3 Laboratory of Ethnomedicine, Parasitology and Drug Discovery, Department of Biological Sciences, College of Science, Technology, Engineering and Mathematics, Alabama State University, Montgomery, AL 36104 USA; 4 Frederick National Laboratory for Cancer Research, National Institute of Health, Frederick, MD 21702 USA

**Keywords:** Catechin gallate, *T. gondii*, Metabolomics, Lipidomics, In vitro

## Abstract

**Background:**

*Toxoplasma gondii* is a zoonotic parasite, the causative agent of toxoplasmosis, which has global importance owing to its significant socioeconomic, public health, and veterinary burdens. Toxoplasmosis is currently treated with a combination of pyrimethamine and sulfadiazine. These drugs have treatment failures and toxicity and are ineffective against the bradyzoite stage. Hence, there is a need for new inhibitors against *T. gondii.* Catechin gallate (CG) is a known antioxidant with demonstrated antiparasitic properties. However, little is known about its anti-*Toxoplasma gondii* activity and mechanism of action.

**Methods:**

Here, we assess the effect of CG on human telomerase reverse transcriptase immortalized foreskin fibroblast (hTERT) cells, cytotoxicity, and inhibitory activity of the RH-RFP (type I) strain of *T. gondii* tachyzoite. Inhibitory and cytotoxicity activities were measured by a fluorescent plate reader, and the data were analyzed using Graph Pad Prism software. In addition, to predict the possible mechanism of CG action, hTERT cells were cultured in a T25 flask and infected with RH-RFP parasites, followed by CG administration and incubation for 48 h. Parasites were quenched under ice, and the parasites were purified from host cells and extracted with chloroform–methanol. The extracts containing the lipids and metabolites were analyzed using liquid chromatography–mass spectrometry (LC–MS).

**Results:**

To address this research question, we tested the in vitro inhibitory activity of CG against parasite growth at 48 h and 72 h. The half-maximal inhibitory concentration (IC_50_) values against tachyzoite growth were calculated to be 10.07 (8.31–12.20) µM and 7.057 (5.98–8.32) µM for 48 h and 72 h, respectively. We identified 5-formyl-tetrahydromethanopterin; 5-(6-hydroxy-6-methyloctyl)-2,5-dihydrofuran-2-one; trans-3-indoleacrylic acid; 5,5-dimethyl-2-{[(2-phenylacetyl)amino]methyl}-1,3-thiazolane-4-carboxylic acid; 5′-*S*-Ethyl-5′-thioadenosine; l-Norleucine; and norepinephrine sulfate as the most produced during the CG treatment. For the lipidomics analysis, we identified the production of several sphingolipid species, including ceramides, dihydroceramide, and sphingosine, which are associated with apoptosis and autophagy. The limited number of sphingomyelin and sphingosine-1-phosphate identified, which are known to promote proliferation, suggests that CG may be affecting *T. gondii* parasites’ proliferation. In addition, oxidized fatty acids (3-hydroxypropyl stearate and (R)-3-hydroxy myristic acid) were observed in both treatments with low production, which confers oxidative stress induction on parasites.

**Conclusions:**

The study showed that CG had inhibitory activity against *T. gondii* growth and caused metabolite and lipid alterations in *T. gondii.* This requires future studies on the enzymes associated with the biosynthesis of these metabolite/lipid pathways that are altered in these in vitro studies.

**Graphical abstract:**

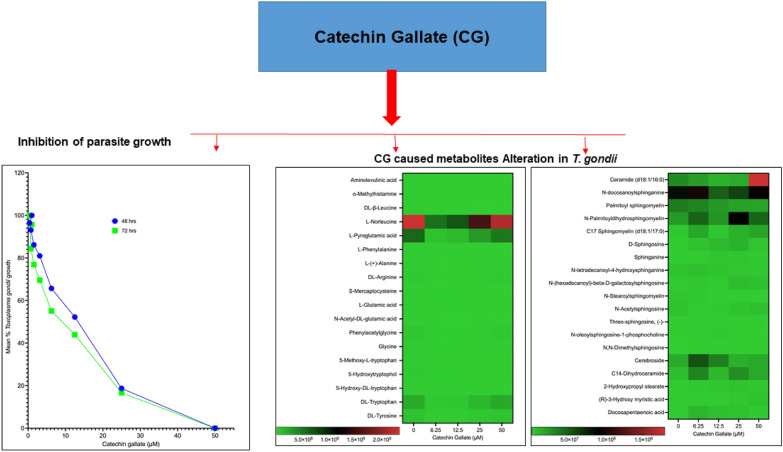

## Background

*Toxoplasma gondii* is a globally prevalent intracellular parasite estimated to infect approximately one third of the world’s population [1]. The parasite can infiltrate and replicate within various nucleated cells [2, 3]. Current anti-toxoplasma medications, namely pyrimethamine (PY), sulfadiazine (SZ), or their combination, showed limited efficacy against toxoplasmosis. These drugs are not effective in eliminating the encysted form of the parasite and often lead to severe toxicities and undesirable side effects, highlighting the need for better-tolerated alternatives [2–4]. Toxoplasmosis, particularly in cases of congenital infection, can have adverse consequences for fetal health, potentially resulting in fetal demise and severe neurological and ocular complications [4, 5]. In addition, individuals with compromised immune systems face life-threatening conditions, such as encephalitis and mortality, due to fresh infections or the reactivation of pre-existing toxoplasma cysts [6]. The uncontrolled progression of *toxoplasma* infections, primarily through the lytic cycle, which involves attachment, invasion, intracellular replication, gliding motility, and egress, can lead to dire and often fatal outcomes [2, 7].

Catechin gallate (CG) is a type of flavonoid found in small amounts in green tea and is known for its various biological activities [8]. The primary sources of catechins are *Camellia sinensis* and *Camellia assamica*, with catechins making up over 75% of the polyphenol compounds in tea leaves [9]. CG inhibits cyclooxygenase (COX) enzymes, which have roles in oxygenating and oxidizing substrates. Blocking Cox-1 and Cox-2 helps alleviate inflammation, including fever, thrombosis, neurodegenerative diseases, and cancer [10, 11]. Given the lack of effective vaccines against parasites, specifically *T. gondii* in humans, modern strategies using ligand- and target-based approaches, new adjuvants, and carriers are promising for employing flavonoids such as catechins to combat parasitic infections. Monomeric catechins show extensive antiprotozoal activity, targeting crucial enzymes in redox and energy metabolism while being selective for host analogs [12]. Oligomeric catechins are cytotoxic to protozoa and helminths, impairing the motility and development of ruminant and bovine nematodes [12]. Catechins offer a range of benefits, including antimicrobial, antiviral, antiinflammatory, anti-allergenic, and anticancer properties. They enhance the efficacy of functional foods and biocosmetics [13]. Moreover, antioxidant compounds from food and plant residues have been demonstrated to be safe for human use [8, 9, 14]. The efficacy of catechins has also been demonstrated in tissue biopsy culture models and in vivo experiments, confirming their safety for human use. Studies have shown that green tea extracts can significantly reduce the levels of *Streptococcus* mutants in children’s saliva and dental plaque [15]. Green tea extracts also possess anti-melanogenic properties in melanocytes, with fermented tea leaves exhibiting the lowest cytotoxicity and highest anti-melanogenic activity [16]. Catechins protect the skin from ultraviolet (UV) rays due to their high antioxidant activity, though they are unstable in sunlight; enhancing the stability of catechins in sunlight can improve their UV protection [9]. The key to improving the stability of catechins lies in addressing sunlight exposure, oxidation, compound stability, and collagen stabilization. Catechins’ antioxidant properties are also applied in dyes, packaging materials, nanoparticles, and biocompatibility [17]. Chemical modifications, molecular interactions, hydrogen bonding, and nanoparticle treatments enhance their efficiency through synergistic effects during extraction and processing. These findings not only highlight the diverse applications of CG but also instill confidence in its safety and efficacy for human use [9, 17]. Here, we investigated its anti-*T. gondii* properties and its effect on *T. gondii* tachyzoite metabolite and lipid production.

## Methods

### Compounds

Catechin gallate was obtained from Sigma Aldrich and dissolved in 0.1% DMSO. CG concentrations used were 6.25; 12.5; 25; and 50 μM for metabolomics and lipidomics analyses.

### Host cells and parasite maintenance

Human telomerase reverse transcriptase immortalized foreskin fibroblasts (hTERT) were obtained from Distinguished Professor Silva NJ. Moreno at The University of Georgia, Athens, GA, USA. hTERT cells were maintained in normal culture medium, Dulbecco’s modified Eagle medium (DMEM); supplemented with 5% (v/v) fetal bovine serum (FBS), 200 nM l-glutamine, and 1% (v/v) penicillin–streptomycin from Life Technologies, USA; and incubated at 37 °C with 5% CO_2_ and 95% atmospheric air. *T. gondii* tachyzoite (type 1 strain), RH-RFP expressing red fluorescent protein in culture, was provided by Dr. Silvia NJ. Moreno from The University of Georgia, Athens, GA, and maintained in hTERT cells.

### Inhibition of intracellular RH-RFP tachyzoite growth

hTERT cells (1.0 × 10^4^ cells/mL) were counted and seeded into 96-well plates/100 µL and incubated at 37 °C supplemented with 5% CO_2_ to grow for 24 h. At 24 h, wells were washed with 1 × phosphate-buffered saline (PBS) twice, and 100 µL of RH-RFP (1.0 × 10^3^ cells/mL) tachyzoite was added to the same 96-well plates and allowed to infect host cells for 3 h. Extracellular parasites were washed off twice with 1 × PBS followed by the addition of drugs serially diluted using procedures published by Huffman et al. [19] and Costa et al. [18]. A volume of 100 µL of CG (0; 0.39; 0.78; 1.56; 3.125; 6.25; 12.5; 25; and 50 µM), positive controls (SZ and PY with concentrations of 0; 3.13; 6.25; 12.5; 25; and 50 µM) as referenced from Huffman et al. [19], and negative control (culture media) were added to the designated wells. Plates were read at 48 h and 72 h using a Tecan 200 F infinite microplate reader, set at an excitation of 560 nm and an emission of 630 nm. The gain was set to 100% optimal with 25 flashes, as described by Huffman et al. [19]. The percent of parasite growth was calculated using the formula reported in Huffman et al. [19], using Graph Pad Prism software version 9.4.1.

### Extraction of metabolites and lipids

For the metabolomics and lipidomics analysis, human telomerase reverse transcriptase immortalized foreskin fibroblast (hTERT) cells (1 × 10^4^) were grown in a T25 flask and allowed to grow to 90% confluency. The flasks were removed from the 37 °C incubator and the media aspirated out, followed by the addition of 3 mL of freshly prepared culture media [5% FBS with 1% penicillin–streptomycin (PS)]. Next, *T. gondii* RH-RFP type strain I (1 × 10^3^ tachyzoite)/50 µL was added to host cells, followed by the addition of 6.25; 12.5; 25; and 50 µM of CG. At 48 h of drug exposure, the hTERT cells and RH-RFP parasites were quenched using ice, then scraped and passed through a 22/26/25-gauge needle, syringed, and filtered via a 3 µL Whatman filter into 50 mL tubes. The parasite suspension was centrifuged at 12 °C for 10 min at 500*g*. The supernatant was removed, and a 2:1 mixture of chloroform:methanol was added to 1 × 10^5^ parasites/tube and vortexed for 5 min using a vortexer model from Fisher Scientific and centrifuged at 12 °C using an Eppendorf centrifuge model 5430 R for 10 min at 500*g*. Approximately 500 µL of chloroform:methanol parasite metabolites or lipids were pipetted into each gas liquid chromatography (GLC) vial and transported under ice to the liquid chromatography–mass spectrometry (LC–MS) facility for analysis.

### LC–MS analysis

Metabolites and lipids were analyzed using a Vanquish UHPLC system from Thermo Fisher, USA, connected to a quadrupole orbitrap mass spectrometer, specifically the Orbitrap Exploris 120 model from Thermo Fisher. This setup employed electrospray ionization (H-ESI) in both positive and negative modes, managed by Xcalibur software (version 4.4.16.14). For the analysis, 10 μL of parasite lipids/metabolites was introduced onto a C18 column (ACQUITY UPLC® BEH C18, 1.7 µM, 2.1 × 50 mm, Waters). The mobile phase consisted of solution A (0.1% formic acid in a mixture of 50% water and 50% methanol) and solution B (a blend of 50% acetonitrile and 50% isopropanol with 0.05% formic acid), delivered at a flow rate of 200 μL/min. The gradient started at 30% B, increased to 50% B within 1 min, followed by a linear progression to 100% B over 13 min, maintained for 3 min, then returned to 30% B for a 3-min re-equilibration period, resulting in a total runtime of 21 min. The spray voltage was adjusted to 3.5 kV in positive mode and 3.0 kV in negative mode. CG injections were performed separately for each mode. Gas settings included a sheath gas of 30, aux gas of 20, and sweep gas at 0 (all in arbitrary units), while the vaporizer and ion transfer tube temperatures were set to 300 °C and 350 °C, respectively. The orbitrap resolution was set at 120,000 for MS and 15,000 for data-dependent acquisition (DDA) MS/MS, with four dependent scans, an intensity threshold of 20,000, automatic dynamic exclusion, and a targeted exclusion mass list based on blank injections. EASY-IC was activated for the MS scan within the range of 115–1000 Da. Collision energy was normalized and stepped at 10, 40, and 100, with the maximum injection time set to automatic. Metabolites and lipids analyses were conducted at the Auburn University Research Mass Spectrometry Facility in Auburn, Alabama. Identifications of metabolites and lipids were carried out using lipid/metabolite standards coupled with the host/pathogens metabolome database and lipid maps.

### Statistics

The heat maps were obtained using GraphPad Prism software version 9.4.1. CA, USA. Means were compared using Tukey’s multiple comparison test, with the alpha value set to 0.05. A scale of 1 × 10^1^ to 3 × 10^9^ was used to group the compounds from not produced to highly produced.

## Results

### Catechin gallate inhibited *Toxoplasma gondii* RH-RFP tachyzoite growth

**Fig. 1:**
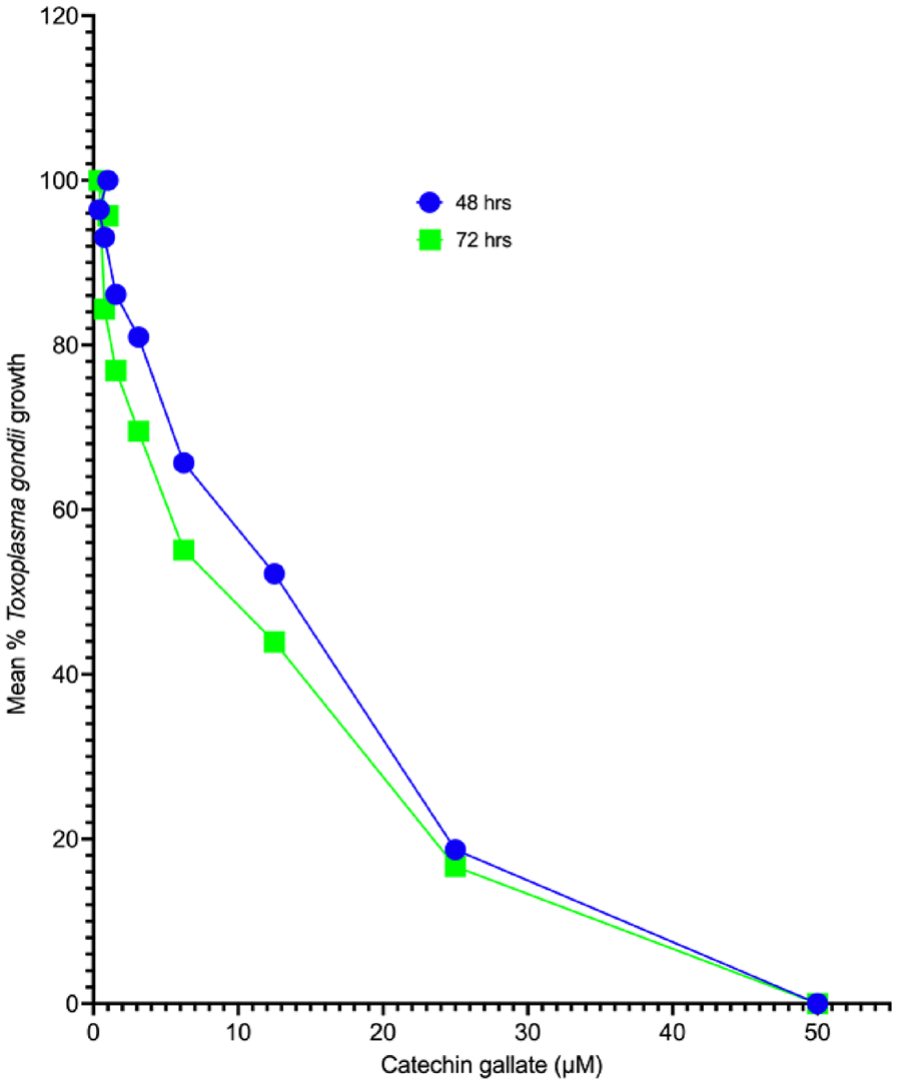
Mean growth curve of *Toxoplasma gondii* tachyzoites in vitro at 48-h and 72-h interaction

We assessed the anti-*T. gondii* activity of CG and compared its effect with the standard drugs, SZ and PY, in vitro, referenced from Huffman et al. [19]. The 50% minimum inhibitory concentration (IC_50_) values after 48 h and 72 h of interaction between CG and parasites were 10.07 (8.316–12.20) µM and 7.057 (5.987–8.326) µM, respectively. The IC_50_ values for SZ and PY after the same times of interaction were 1.55 and 0.95 µM for SZ and 3.52 and 2.42 µM for PY at 48 h and 72 h, respectively. Surprisingly, the effectiveness of CG was greater at 72 h compared with the IC_50_ value obtained at 48 h. Similarly, SZ and PY were more effective at 72 h than at 48 h. The growth curves for CG have been represented in Fig. 1.

### Metabolites induced by catechin gallate

**Fig. 2:**
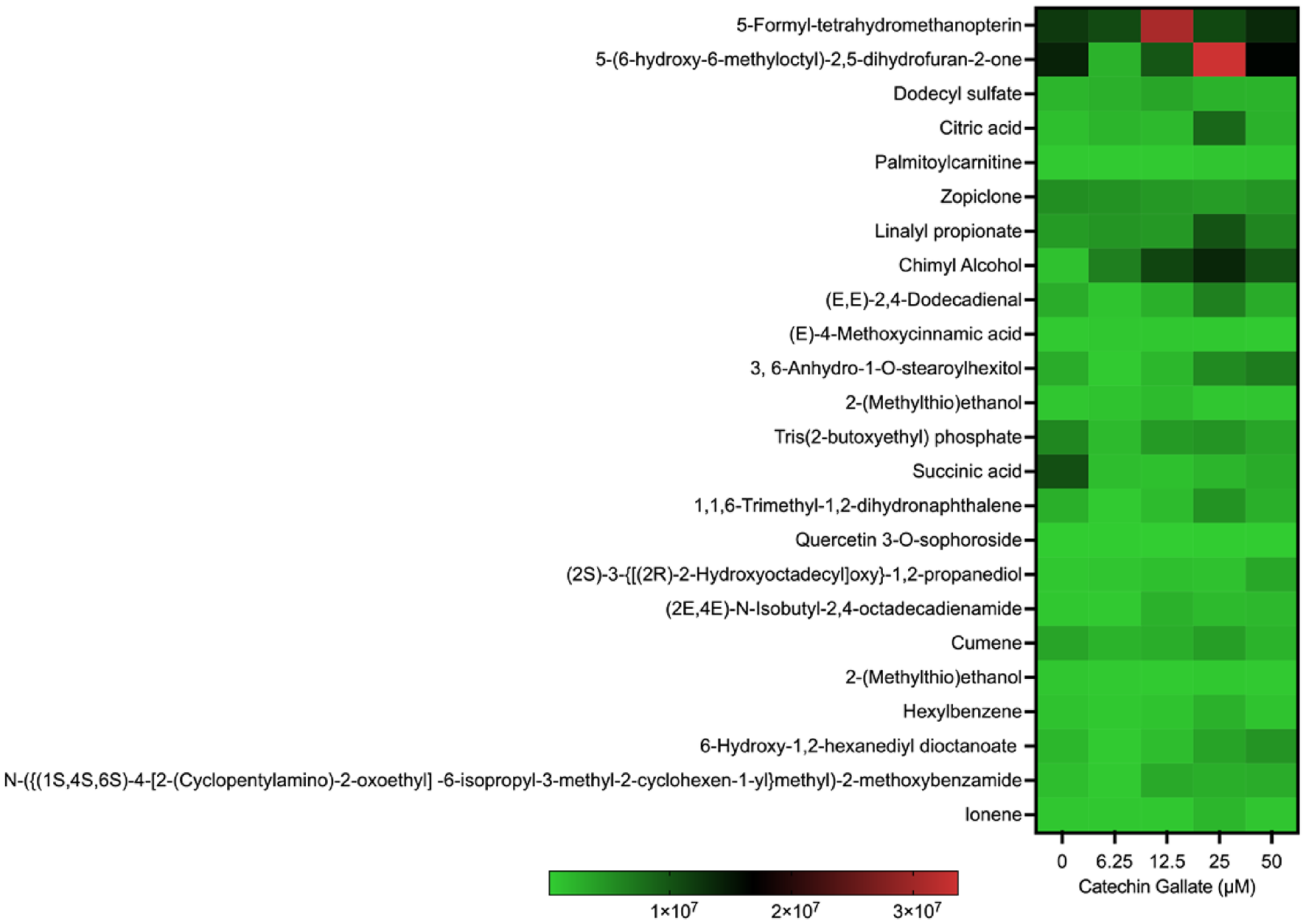
Heat map showing the distribution of metabolites produced by CG treatment on *Toxoplasma gondii* tachyzoites in a concentration-dependent (6.25, 12.5, 25, and 50 µM) manner at 48 h

**Fig. 3:**
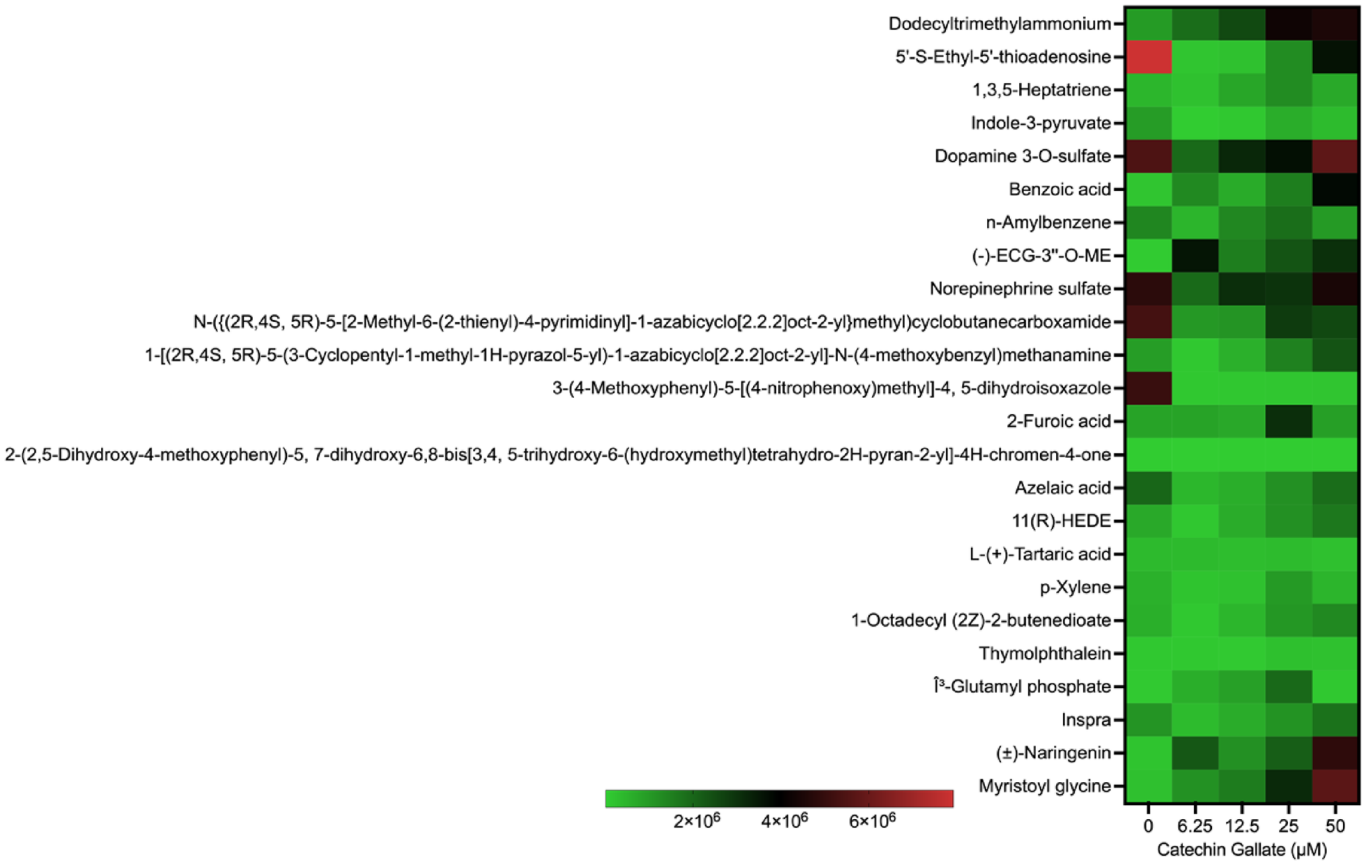
Heat map showing the distribution of *Toxoplasma gondii* tachyzoites metabolites elicited by CG treated in a concentration-dependent (0, 6.25, 12.5, and 50 µM) manner at 48 h

To assess the effect of CG on *T. gondii* tachyzoite metabolite production, LC–MS analysis was conducted. Interestingly, a total of 1639 metabolites were identified. Among these, 48 metabolites showed alterations with a *P*-value < 0.05 in both the control and treated groups. Specifically, 11 of these metabolites exhibited significant differences with *P*-values less than 0.05 when treated with CG. Some of the identified metabolites included palmitoylcarnitine; chimyl alcohol; Tris(2-butoxyethyl) phosphate; dodecyltrimethylammonium; Indole-3-pyruvate; benzoic acid; (−) ECG-3″-*O*-ME; *N*-({(2R,4S,5R)-5-[2-methyl-6-(2-thienyl)-4-pyrimidinyl]-1-azabicyclo [2.2.2] oct-2-yl} methyl) cyclobutane carboxamide; 3-(4-methoxyphenyl)-5-[(4-nitrophenoxy) methyl]-4,5-dihydroisoxazolem; (±)-Naringenin; and Myristoyl glycine. The 48 metabolites with significant statistical relevance are shown in Figs. 2 and 3.

Notably, 5-Formyl-tetrahydromethanopterin and -(6-hydroxy-6-methyloctyl)-2,5-dihydrofuran-2-one were slightly produced during CG treatments at 12.5, 25, and 50 μM with a scale ranging between 1 × 10^7^ and 3.0 × 10^7^ (Fig. 3). The remaining compounds were less produced in both treated and control groups as indicated in (Fig. 3).

### Catechin gallate induces sphingolipid production

**Fig. 4:**
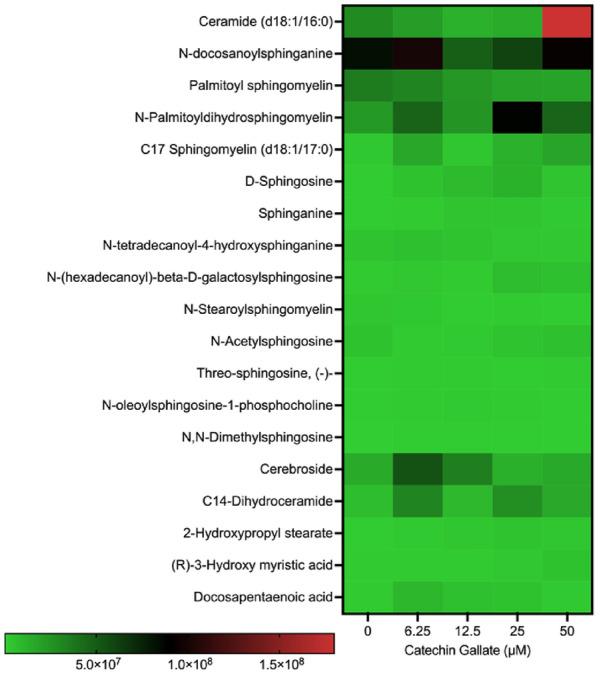
Heat map showing the distribution of sphingolipids and fatty acids produced by *Toxoplasma gondii* when treated with CG in a concentration-dependent (0, 6.25, 12.5, 25, and 50 µM) manner at 48 h

We detected 15 sphingolipid species produced when CG was treated on the tachyzoites. The most common ones were the ceramides, sphingosine, sphingomyelins, and sphinganine (Fig. 4). Sphingolipids such as ceramide, *n*-docosanoylsphingoganine, *n*-palmitoyldihydrosphingomyelin, cerebroside, and C14-dihydroceramide were moderately produced at either 6.25, 12.5, 25, and 50 μM treatment with CG.

Ceramide was produced at a lower concentration in the control groups but was highly produced at 50 μM, with a scale intensity above 1.5 × 10^8^ (Fig. 4). *N*-Docosanoylsphinganine was moderately produced at the control and 6.25 μM (with a scale intensity below 1.0 × 10^8^). Similarly, we observed the same pattern at 12.5 μM treatment. Contrarily, the same sphingolipid was moderately formed at 25 μM and 50 μM treatment with CG (with a scale intensity below 5.0 × 10^7^). In addition, *N*-Palmitoyldihydrosphingomyelin was observed to be moderately produced at 25 μM (with a scale intensity below 1 × 10^8^). Moreover, cerebroside and C14-dihydroceramide were also moderately produced in parasites at 6.25 μM treatment with a scale intensity above 5 × 10^7^.

### Amino acid and peptide production

**Fig. 5:**
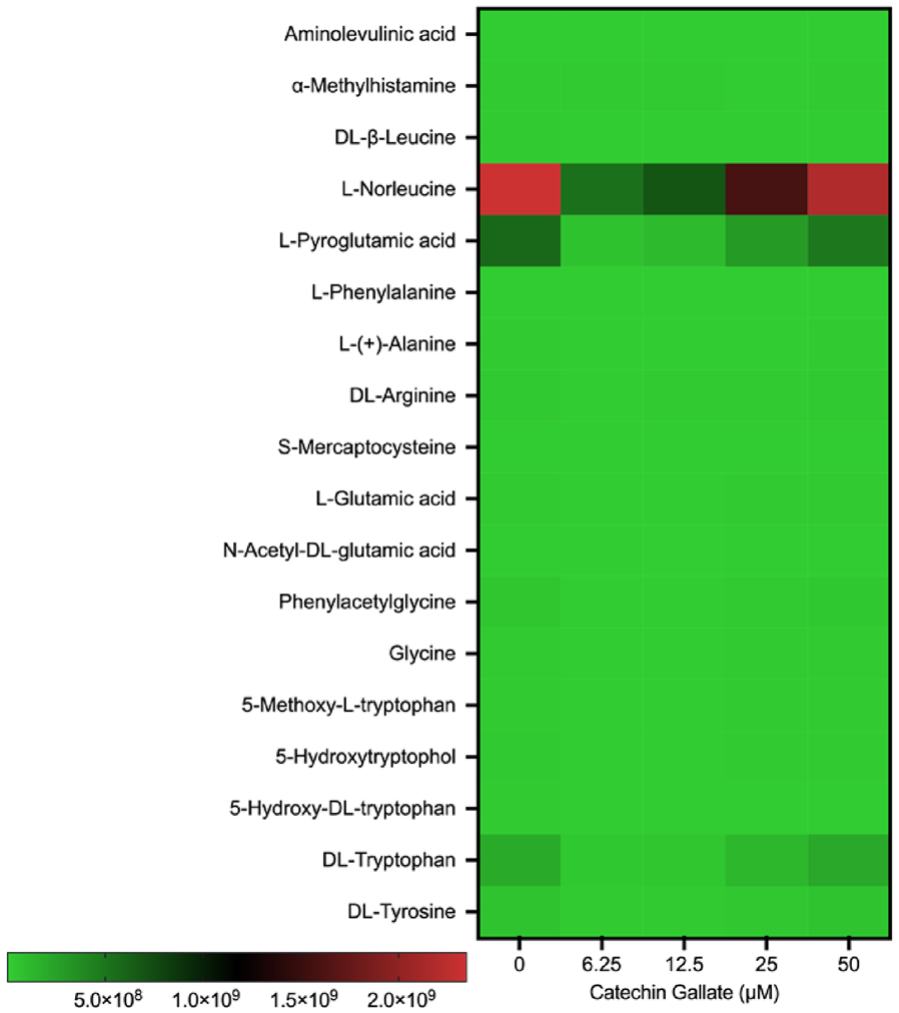
Heat map showing amino acid production in *T. gondii* tachyzoites treated with CG in a concentration-dependent (0, 6.25, 12.5, 25, and 50 µM) manner at 48 h

**Fig. 6:**
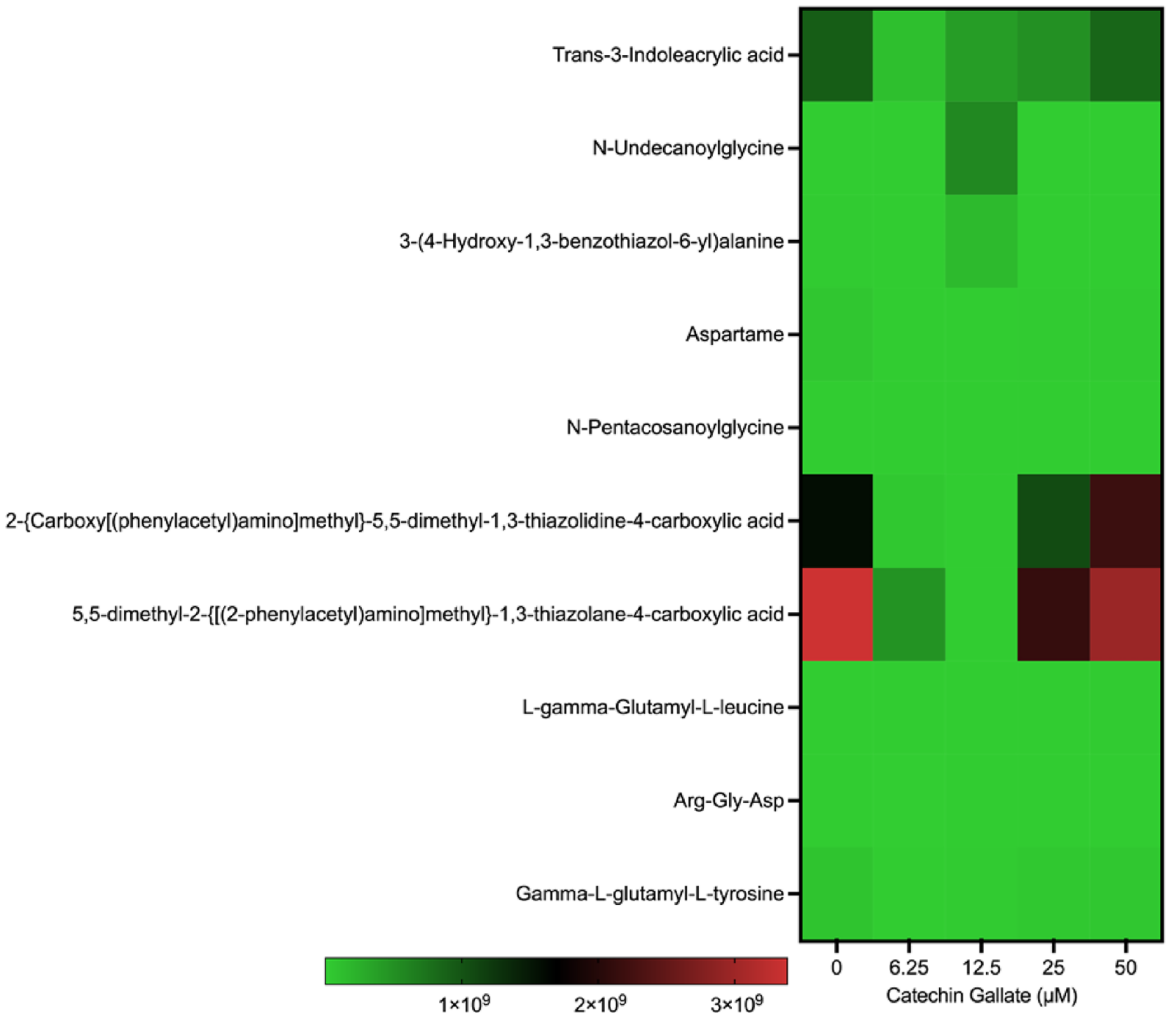
Heat map showing peptide and non-peptide production in *Toxoplasma gondii* tachyzoites treated with CG in a concentration-dependent (0, 6.25, 12.5, 25, and 50 µM) manner at 48 h

In addition, we found certain amino acids and their derivatives as part of the metabolite profile. The amino acid detected with a slight increment was the l-norleucine at different concentrations of CG (Fig. 3A). Some of the amino acids were methylated, phenylated, acetylated, hydroxylated, and methoxylated (Fig. 5). Interestingly, some of the amino acids were modified through acetylation, methylation, methoxylation, methylation, hydroxylation, and phenylation (Fig. 5). These amino acids were less expressed. Moreover, we detected peptides and non-peptides as summarized in Fig. 6. Trans-3-indoleacrylic acid and *N*-Undecanoylglycine were moderately formed in the control group, at 25 μM and 50 μM treatment with CG treatment with scale intensities around 2 × 10^8^ and 3 × 10^8^ (Fig. 6).

### Phosphocholine (PC) species production

**Fig. 7:**
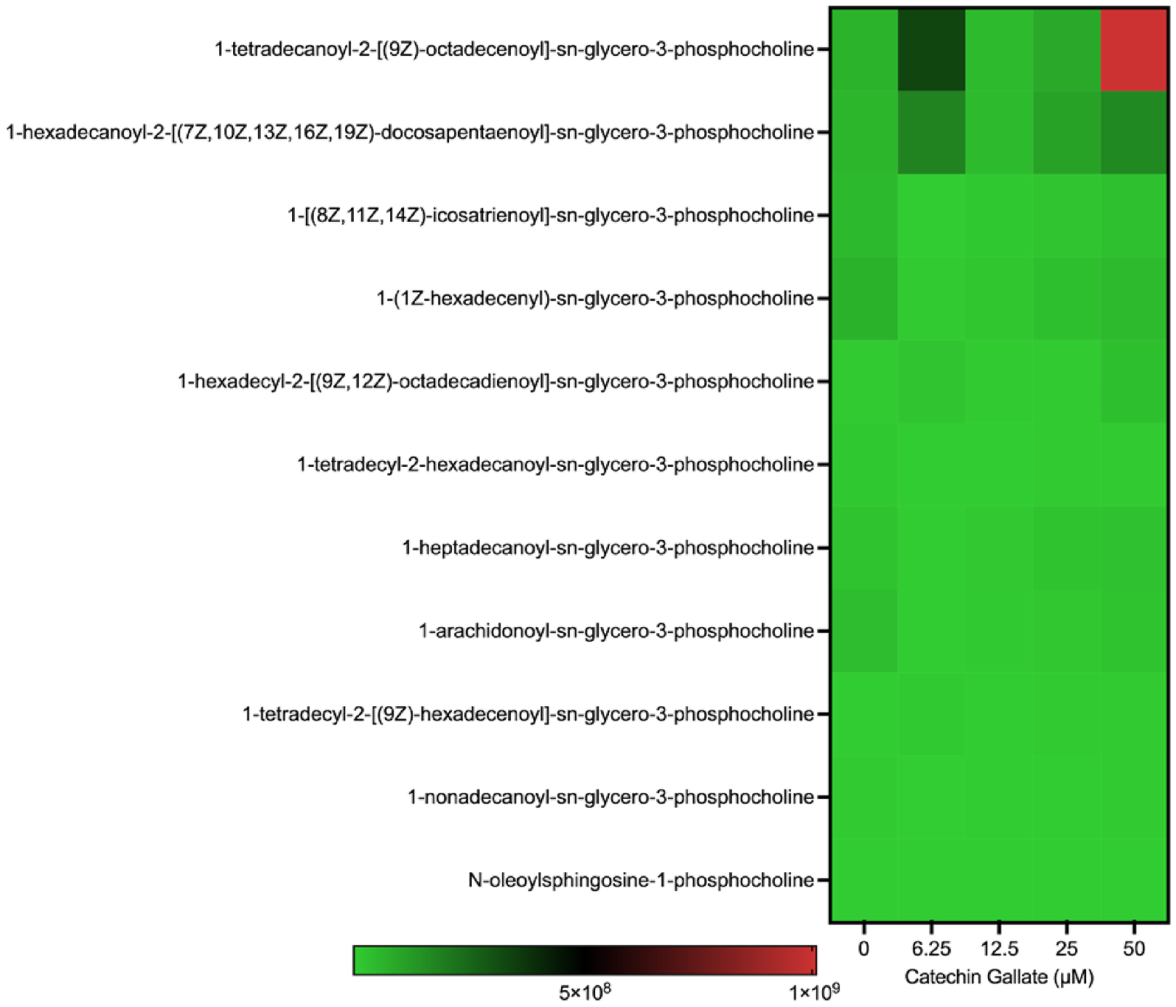
Heat map showing phosphocholine production in *Toxoplasma gondii* tachyzoites treated with CG in a concentration-dependent (0, 6.25, 12.5, 25, and 50 µM) manner at 48 h

We detected 11 phosphocholine (PC) species in both treated and untreated parasites (Fig. 7). The PC species that was highly produced at 50 μM was the 1-Tetradecanoyl-2-[(9Z)-octadecenoyl]-*sn*-glycero-3-phosphocholine with a scale intensity of 1 × 10^9^). Every other phosphocholine species produced was less expressed with a scale intensity below 2 × 10^8^ (Fig. 7).

### Phosphoethanolamine (PE) species production

**Fig. 8:**
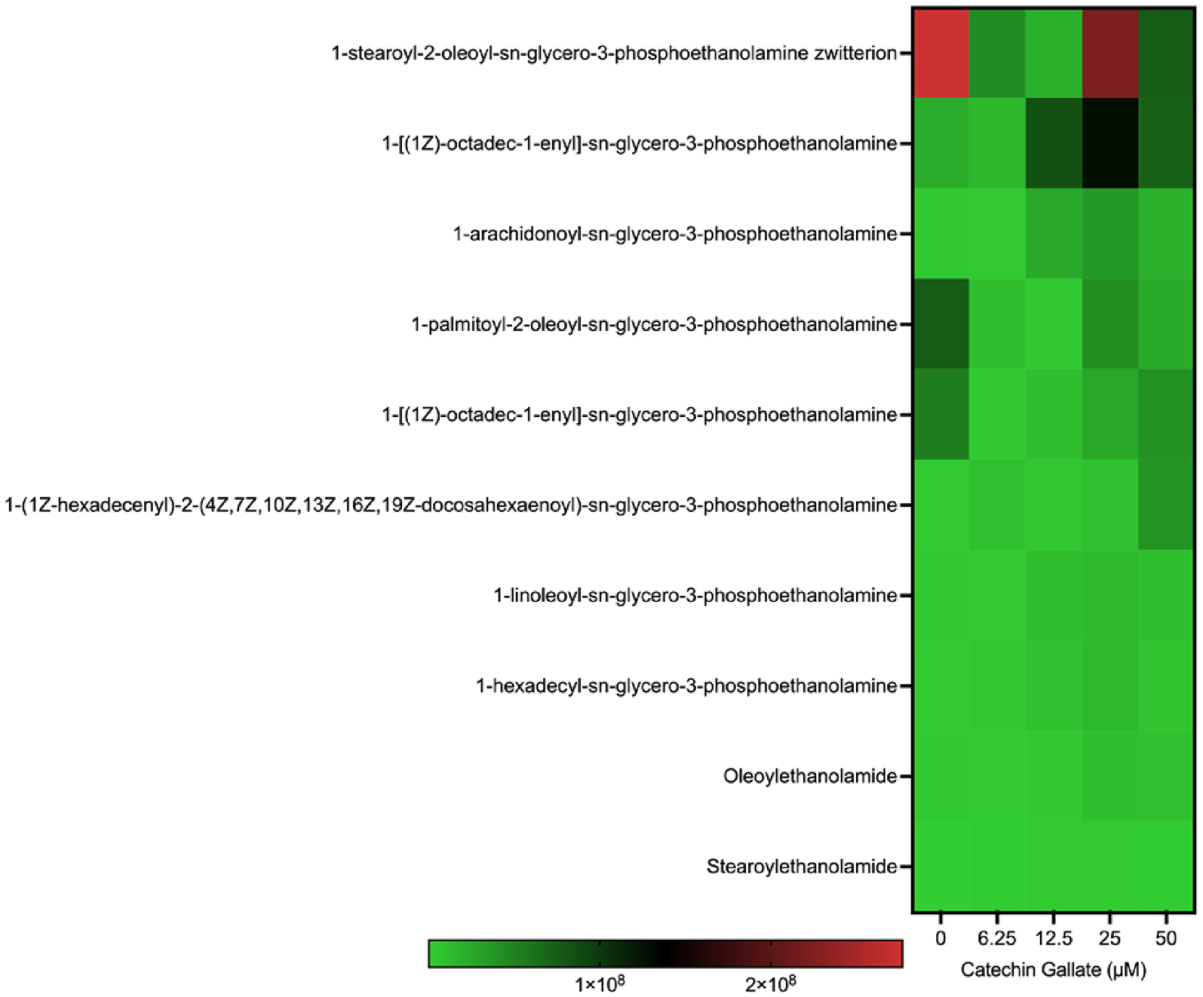
Heat map showing phosphoethanolamine production in *Toxoplasma gondii* tachyzoites treated with CG concentrations (0, 6.25, 12.5, 25, and 50 µM) at 48 h

Phosphoethanolamine species were also detected in all treatments and the control parasite group (Fig. 8). PE species 1-Stearoyl-2-oleoyl-*sn*-glycero-3-phosphoethanolamine zwitterion was highly expressed at the control and 25 μM (with a scale intensity above 2 × 10^8^) but not in the other concentrations used. Another PE detected was 1-[(1Z)-octadec-1-enyl]-*sn*-glycero-3-phosphoethanolamine. The production of this PE species was lowly expressed at the control, 6.25, 12.5, and 50 μM treatment groups (with a scale intensity between 1 × 10^8^ and 1.5 × 10^8^). However, at 25 μM treatment with CG, it was moderately expressed (with a scale intensity closer to 2 × 10^8^). Whereas the remaining eight phosphoethanolamine species were lowered in formation throughout the concentrations (below 1 × 10^8^) used (Fig. 8).

## Discussion

Toxoplasmosis is increasing globally, with about one third of the human population having experienced *T. gondii* infection, and remains a significant public health challenge in humans and animals [1]. Presently, in the USA, the first line of treatment of toxoplasmosis is through a combination of pyrimethamine, sulfadiazine, and folic acid [20, 21]. Although this combination has some efficacy against the tachyzoite form, it is associated with toxicity and ineffective against the bradyzoite stage [21–23]. These drawbacks necessitate the search for new inhibitors against *T. gondii* for future development against toxoplasmosis.

In this study, catechin gallate (CG) was tested against *T. gondii* tachyzoite using the RH-RFP reporter (type I) strain. The IC_50_ values obtained were within the ranges reported in other natural products and synthetic compounds discovered to have anti-*T. gondii* activity by other authors [24–26]. Notably, the IC_50_ values of CG at 48-h and 72-h interactions with parasites in vitro were less effective than the current drugs, SZ and PY, used as positive standards referenced from Huffman et al. [19]. This implies that using it alone may not be effective unless medicinal chemistry optimization is carried out in future studies. Our data on this CG disagreed with previous studies of other dietary polyphenols reported against other *Leishmania* and other parasites [27].

Polyphenol compounds have been reported in plants and are essential to protecting tea leaves against pathogens; in addition, they have antiparasitic effects [28]. Catechins are known as potent antioxidants. Hellmann et al. mentioned that epigallocatechin catechin affects sporozoites’ gliding motility in *Plasmodium* spp. [9]. It has been reported that an oxidative stress inducer causes apoptosis; an increase in calcium influx; oxidized fatty acids, sphingolipids, ceramides, and sphingomyelins; DNA oxidation, nitrative modification; and toxic organic compounds [29–31]. Our study discovered that the highest concentration of CG resulted in some toxic organic compounds (Fig. 3). These observations support previous studies that showed that oxidative stressors could induce toxic organic compound formations and their possible interaction with lipids, proteins, and nucleic acids, and eventually cell death [29–36].

Sphingolipids play diverse roles in cellular processes, including cell membrane structure and function, signal transduction, and cellular signaling pathways. For example, sphingosine and sphinganine are involved in cell signaling pathways regulating cell growth, differentiation, and apoptosis (programmed cell death) [37]. Sphingomyelins are significant components of cell membranes and are implicated in membrane integrity and lipid metabolism [38, 39]. The specific functions of each sphingolipid can vary on the basis of its chemical structure and interactions within cellular pathways, highlighting the complexity of sphingolipid biology in cellular physiology and pathology [38, 39]. Ceramides and sphingosine are known to be associated with apoptosis in cells [40]. In this study, we detected ceramides and many kinds of sphingosine, which implied that the inhibitory activity of CG could be associated with parasite apoptosis. Furthermore, it has also been reported that dihydroceramide causes autophagy [40]. Thus, the few dihydroceramides produced by the parasites could have caused the parasites to undergo autophagy. Future studies will be needed to ascertain this conjecture.

Another interesting finding was the formation of hydroxyl fatty acids, which are known to be caused by oxidative stress (reactive oxygen species and superoxide imbalance in the cell), leading to lipid peroxidation. Moon et al. [29] reported that a natural compound (apigeninidin chloride) caused lipid-oxidized compounds formation in *T. gondii* tachyzoite and could partly induce tachyzoite death. We believe that the fewer hydroxyl fatty acids((R)-3-hydroxy myristic acid and 2-hydroxypropyl stearate)) formed could have been toxic to tachyzoites, resulting in parasite death [29, 41].

The modification of amino acids through methylation, methoxylation, phenylation, hydroxylation, acetylation, and myristylation (Fig. 5) obtained could imply that CG might affect parasite protein synthesis via a direct or indirect manner. Several authors have reported the effect of natural products and synthetic compounds that cause oxidative stress and induce protein modifications [29, 41, 42]. Thus, further studies are required to ascertain this assumption using a proteomics approach.

Phosphocholine, a phosphorylated form of choline, is a vital component of cellular membranes and serves various crucial functions in cells and parasites such as *T. gondii*. Phosphocholine is fundamental in maintaining membrane integrity and fluidity, facilitating cellular communication, and serving as a precursor for phosphatidylcholine synthesis. Charron and Sibley [43] proposed that the parasite can quickly obtain phosphatidylcholine from the host cell. Our research showed that only a few PC and PE species were being slightly produced, suggesting that CG might have inhibited the membrane phospholipid biosynthesis. However, since the study was a whole lipidomics analysis, we suggest that future studies focus on enzymatic pathway analysis to decipher the enzymes inhibited by CG, leading to the low expression of this lipid species.

## Conclusions

Our study showed that CG has antiparasitic activity in vitro and affects metabolites and lipid synthesis in *T. gondii* tachyzoites. Specifically, it caused oxidized lipids, amino acid modification, and apoptotic sphingolipid biosynthesis according to our LC–MS data. Therefore, these lipids, modified amino acids, and organic toxic compounds produced could have caused parasite apoptosis and autophagy activities, leading to possible parasite death. Further studies will be needed to examine the effect of CG on the enzymes involved in the committed steps in the lipid and amino acid biosynthesis pathways to fully understand CG’s mechanism of action and future potential as an anti-*Toxoplasma* lead for in vivo studies.

## Data Availability

Data supporting the main conclusions of this study are included in the manuscript.
